# Ultra-wideband microstrip folded antenna for wireless LAN, 5G and Internet of Things applications

**DOI:** 10.1038/s41598-024-65502-6

**Published:** 2024-11-26

**Authors:** Utkarsh Pandey, Parulpreet Singh, Raghvendra Singh, Vivek Kumar, Kanad Ray, Saurav Mallik, Haya Mesfer Alshahrani, E. Elshiekh, Mohamed Abbas, Ben Othman Soufiene

**Affiliations:** 1 ECE, Lovely Professional University, Punjab, India; 2 ECE, Pranveer Singh Institute of Technology, Kanpur, India; 3 Amity School of Applied Sciences, Amity University Rajasthan, Jaipur, 303002 India; 4 Amity Cognitive Computing and Brain Informatics Centre, Amity University Rajasthan, Jaipur, 303002 India; 5 Facultad de CienciasFisico-Matematicas, Benemérita Universidad Autónoma de Puebla, Av. San Claudio y AV. 18 Sur, Col. San Manuel Ciudad Universitaria, 72570 Pueble, Pue Mexico; 6 Faubert Lab, Université de Montréal, Ecole d’optométrie, Montréal, QC H3T1P1 Canada; 7 Department of Environmental Health, Harvard T H Chan School of Public Health, Boston, MA 02115 USA; 8 Department of Pharmacology and Toxicology, The University of Arizona, Tucson, MA 85721 USA; 9 Department of Information Systems, College of Computer and Information Sciences, Princess Nourah bint Abdulrahman University, P.O.Box 84428, 11671 Riyadh, Saudi Arabia; 10 Department of Radiological Sciences, College of Applied Medical Sciences, King Khalid University, Abha, Saudi Arabia; 11 Electrical Engineering Department, College of Engineering, King Khalid University, 61421 Abha, Saudi Arabia; 12 PRINCE Laboratory Research, ISITcom, Hammam Sousse, University of Sousse, Sousse, Tunisia

**Keywords:** Energy science and technology, Engineering

## Abstract

This paper presents an ultra-wideband (UWB) microstrip antenna with a simple structure operating at 2.2–6 GHz bandwidth. The proposed antenna has been demonstrated for its suitability in UWB and multi-band applications, such as wireless local area network (WLAN), Wi-Fi, 5G, Internet of Things (IoT), etc. The antenna is shown to use a simple folded dipole antenna structure demonstrating an omnidirectional radiation pattern of gain 0.8 to 6 dBi throughout the frequency band. The antenna has strong gain at 2.4, 3.5 and 5.5 GHz. The measured results are shown to have a good agreement with the simulated results.

## Introduction

The telecommunication industry has been advancing significantly in last decade with the introduction of technologies such as internet of things, self-driving cars, machine-to-machine communication, vehicle to vehicle communication telemedicine and much more. These new technologies have been increasing congestion in the network, raising the need for higher bandwidth, stable network, and faster data rates^1,2^. To meet the requirements being raised by the community, the fifth generation (5G) technology has been introduced to operate at sub-6 GHz band to compliment the already existing sub-3 GHz band^3^ 0.5G is being deployed with the aim to achieve high data throughput, low latency, and high reliability. Microstrip antennas offer a low profile with suitable gain and ease of fabrication, which makes them suitable for applications in base stations as well as mobile devices such as smart phones, smart watches, etc.^4^. They can easily be integrated with the mobile hardware offering further robustness and easy scalability for mass production.

Technologies such as multiple-input-multiple-output (MIMO) and beamforming are being explored extensively in the literature to introduce a strong connectivity and enable the new 5G technology^5^. However, there is a slight lack in technological advancements in the development of antennas for smart hand-held devices, which is being explored very significantly. Several mobile manufacturers such as Samsung and Huawei have started establishing testbeds and have started to come up with several new smart phones that support 5G network^6–8^. Such phones use a variety of antennas such as microstrip patch arrays^9–11^, monopole antennas^12^, dielectric resonator antennas^13,14^, etc. Such antennas along with the help of advanced methods like MIMO and beamforming can help improve the channel capacity and data-rates significantly, making the system more reliable^15^.

Several different antennas have been shown in the literature operating at sub-6 GHz band which offer dual-polarization and good gain. However, because of the complex environment and high electromagnetic noise that we have in the present day, a 5G antenna system is not enough to meet the several different demands of a wireless system which involves the use of Wi-Fi, Wi-Max, LTE, WLAN, Bluetooth, GPS, etc. Hence, a multiband antenna system is required to meet the necessary requirements that can operate on all the suitable frequencies within the sub-6 GHz band. Several multi-band antennas have been proposed in the literature^16–19^ but they tend to have a large size and inadequate current distribution which affects their performance significantly.

One of the proposed antennas for 5G communication, transmits and receives signals at two frequencies having two lower angle of radiations which provides long and better transmission and reception to its nearest base stations for 5G mobile systems. It covers a frequency band of 3.3–3.8 GHz (For upper band) and 4.8–5 GHz (For lower band), it also has a high cross-polar discrimination and high isolation (> = 20 dB) between plates. It is made up of a hollow (cavity) reflector, two double-oval dipoles, and two feeding lines in the shape of an oval. To feed the whole antenna, part of dipole antenna and its one arm is employed as feeding lines which can enhance impedance matching. Performance of dual band is obtained by merging small oval shaped loops into the large oval shaped loops without extending the radiating patch size (0.26λ_o_ × 0.26λ_o_, where λ_o_ is free space wavelength at 3.3 GHz). Antenna gain performance is enhanced by the cavity reflector (7.56 dBi in lower band and 7.42 dBi in upper band) and reduces its size which is only 0.66λ_o_ × 0.66λ_o_ × 0.2λ_o_. Radiation patterns are stable for both bands and its half power bandwidth is within 65° ± 5°. It’s easy fabrication and good agreement with measured and stimulated results makes it a good design for 5G mobile base stations^20^.

One of the prototypes of antenna presents a very economical and a novel broadband base station antenna element, it has a very small size and volume of 14.5 × 14.5 × 3.56 mm^3^. It consists of dual-dipole radiator (Omnidirectional and very effective for Wi-Fi) and an open-box shaped reflector. Dipole of dual hexagon shapes are used to design its each arm by adjusting the gaps, it acts as a main radiating portion and improves bandwidth of the antenna. Four small parasitic patches of hexagonal shapes are also used which decreases the reflection coefficient. Two dipoles are placed orthogonally to excite dual polarizations. Open-box shaped reflectors are made from planar metal plates with “inverted L” shaped edges which are stacked beneath radiators. These reflectors enhance the gain of antenna and reduces the half power bandwidth in 3.3–3.8 GHz. The constructed prototype has a measured 1.68–3.8 GHz of bandwidth, coupling under − 20 dB, steady HPBW with 65° ± 5°, and an average gain of 8.5 dBi. Value of cross-polar discrimination which enhances SNR and measures polarity of antenna is better than 20 dB at boresight and 10 dB within ± 60° directions. Above all explanations conclude that it is a good prototype for 2G/3G/4G/5G base station applications^21^.

One of the design of antennas discussed in literature review is very compact in size as it occupies a space of 0.3λ_o_ × 0.15 λ_o_ × 0.05λ_o_ and its wideband folded antenna provides high input impedance. To increase the input impedance bandwidth, multiple resonant modes are manipulated, shifted and combined by shorting pins, CFS and parasitic patches. For low profile planar folded dipole antenna is used which gives three resonant modes and it is coupled with a feeding structure. In this design two folded dipole antennas are used for good radiation of equal power in all direction (Perpendicular to its axis). It achieves a bandwidth of 80% from 1.57 to 3.68 GHz. Across a wide bandwidth of 82%, it displays flat gain variation with less than 1.27 dB of change in horizontal planes. Thus, it would be good design for wireless access point, micro base stations and indoor signal coverage^22^.

One of the proposed designs of antenna presented in literature review is developed with dual polarized magneto-electric dipole excited by “inverted L” shaped feeding probes for next 5G wireless communications. It consists of four fishtailed shaped patches placed horizontally which plays key role to stabilize far fields and four patches are placed vertically that are shorted to the ground. It operates in the N77/N78 band (often used for lower cellular spectrum) at frequencies between 3.05 and 4.42 GHz with SWR $\le$ 1.5, and its calculated gain ranges from 7.09 to 9.36 dBi with strong front to back ratios. The suggested antenna's impedance bandwidth is 37%. For two polarizations, very stable high-power bandwidth (Frequencies within the bands of interest), stable radiation patterns (Symmetric in nature) and low cross polarizations are observed in both horizontal and vertical planes. All the results conclude that it is a good design of antenna for application to N77/N78 band^23^. For applications in the gastrointestinal tract, this implantable antenna design introduces In-Body to In-Body and In-Body to On-Body Channel Models. The in-body planar ecovercal ring antenna serves as the foundation for the channel models that covers the lower frequency portion of UWB. A semi-circle monopole antenna (Resonant Antenna) with a wideband, operating from 3.1 to 5.1 GHz, is proposed for on-body receivers. Wider frequency ranges may be covered by an elliptical ring of antenna, and UWB is the only frequency band that can deliver high-quality images while simultaneously enabling low output power technology. Its outcomes are confirmed using a wideband on-body monopole antenna within a real UWB liquid phantom. WCE (Wireless Capsule Endoscope) technology has gained popularity in recent years, dispensing with the conventional wired endoscopy that could endanger patients in the medical and surgical fields. WCE is clearly understood by describing the channel inside the lossy medium, where the electric field rapidly declines in the direction of wave propagation, and it also helps in the assessment of its radiation performance. When patients use these wireless capsules, it interacts with various electrically diverse human organs, changing the antenna's resonance frequency. Based on their radiation performance, simulation results and measured reflection coefficients this implanted antenna can be useful for examining channel models within humans as well as in animals in upcoming time^24^. This article describes the modification made in flexible antennas. This antenna consists of Polydimethylsiloxane (PDMS) composite as the substrate, patch and ground planes made of copper (Cu), and coaxial feed using SMA connector. The substrate's dimensions are 60 × 60 × 3 mm^3^, the patch's radius is 21.5 mm, and the ground plane's area is 60 × 60 mm^2^. Copper is a highly good electrical conductor, and PDMS is utilised to create antenna for body-worn applications. In order to improve antenna performance, a PDMS + glass microsphere composite is developed here to replace the PDMS substrate. This composite lowers relative permittivity of the PDMS to 1.9, and loss tangent to 0.014. To maintain a fixed separation from the ground plane and to resolve the adhesiveness issue between PDMS and Cu patch, an additional thin layer of PDMS/PDMS + glass substrate, measuring 0.6 mm in thickness, was used to encapsulate the antenna. Prior to construction, the antenna’s resonance frequency was simulated using CST software. The resonance of PDMS substrate antennas is 1.92 GHz (without encapsulation) and 2.34 GHz (with encapsulation), according to measurements made with a Vector Network Analyzer (VNA), while the resonance of PDMS + glass substrate antennas is 2.46 GHz (without encapsulation) and 2.25 GHz, respectively. The flexible patch antenna's characterisation is carried out by coupling a coaxial cable with a SMA connector to the VNA port S11 (Agilent E5062A). In order to determine if an antenna will resonate inside the ISM frequency band, it is critical to monitor the return loss value of antenna between 300 kHz and 3 GHz. The incident power of 10% will be reflected back to the source if a feedback signal at − 10 dB is received by the antenna. As a result, if the value falls below − 10 dB, the antenna is working well in terms of RF performance because there is minimal return loss. The data from the measurements and results discussed above indicate a suitable fit for the modification of a wearable patch antenna for medical uses^25^.

This proposed paper presents a wideband antenna system operating from 1.8 GHz to 6 GHz, with high efficiency and high gain at 2.4 GHz, 3.6 GHz, and 5.5 GHz, which are suitable for applications in LTE, GPS, Wi-Fi, Wi-Max, and 5G network applications. The antenna is shown to be compact and is suitable for mobile devices. The paper is divided as follows: Section “Antenna design” describes the antenna design, followed by measured and simulated results discussed in section ““Simulated and measured results”” and a conclusion drawn in section IV.

## Antenna design

We started with the targeted frequency band for use which is this 5G communication band (3.2–3.5 GHz). Then we selected the Di electric substrate which is Roger’s 5880 dielectric of 0.787 mm thickness and flexible. By calculating patch dimensions of width and length which got adjusted as per interference later on with simulation done in antenna designing software.

**Figure 1:**
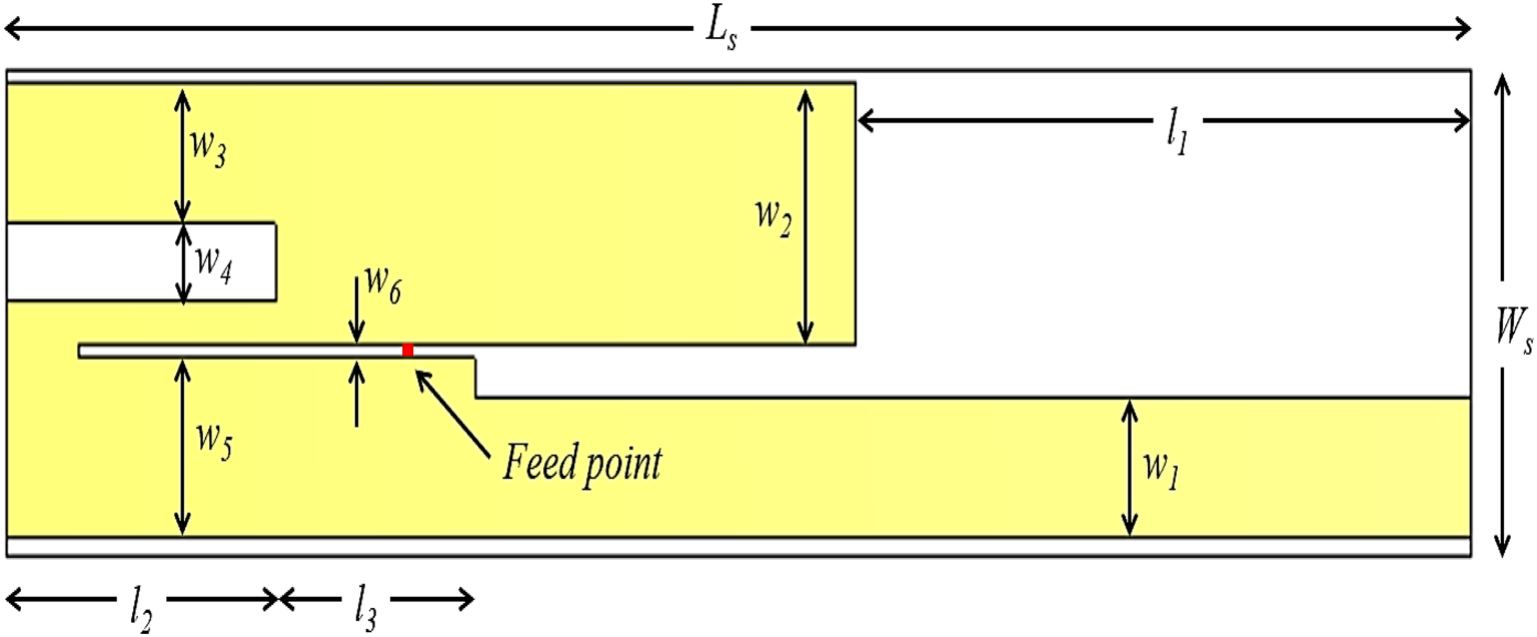
3D model of the proposed antenna design simulated using CST Microwave Studio, dimensions marked in Table 1.

The proposed antenna design is shown in Fig. 1. The proposed antenna is a microstrip folded antenna fabricated on a Rogers RT-5880 substrate of relative permittivity, *ε*_*r*_ = 2.2, loss tangent, *tan δ* = 0.0009 and height, *h* = 0.787 mm. The antenna is 50 mm × 11.3 mm × 0.787 mm (0.5 λ_0_ × 0.113 λ_0_ × 0.008 λ_0_ at 3 GHz) in size.

As we know for folded meander line antenna's resonant frequency components are usually determined by examining its impedance characteristics across a frequency range. Resonant frequency analysis is used to determine the resonant frequencies of a folded meander line antenna. Finding length and path length is the first stage. The resonant frequency of the meander line is determined by its entire effective electrical length. Because of the folded structure, the effective length is frequently longer than the actual length. The following formula for a half-wavelength antenna can be used to approximate the resonant frequency f_r_:

$${\text{f}}_{{\text{r}}} = {\text{c}}/{\text{2L}}_{{{\text{eff}}}}$$

Consider the effects of distributed capacitance and inductance along the folded meander line, which can shift the resonant frequencies from the simple half-wavelength estimation this process has been done through simulation. All the dimensions of the antenna are specified in Table 1.

**Table 1:** Dimensions of the proposed antenna; see Fig. 1.

Label	Value (mm)	Label	Value (mm)
*L*_*S*_	50.0	*w*_*2*_	6.1
*W*_*S*_	11.3	*w*_*3*_	3.3
*l*_*1*_	21.0	*w*_*4*_	1.8
*l*_*2*_	9.2	*w*_*5*_	4.1
*l*_*3*_	6.8	*w*_*6*_	0.3
*w*_*1*_	3.3		

**Figure 2:**
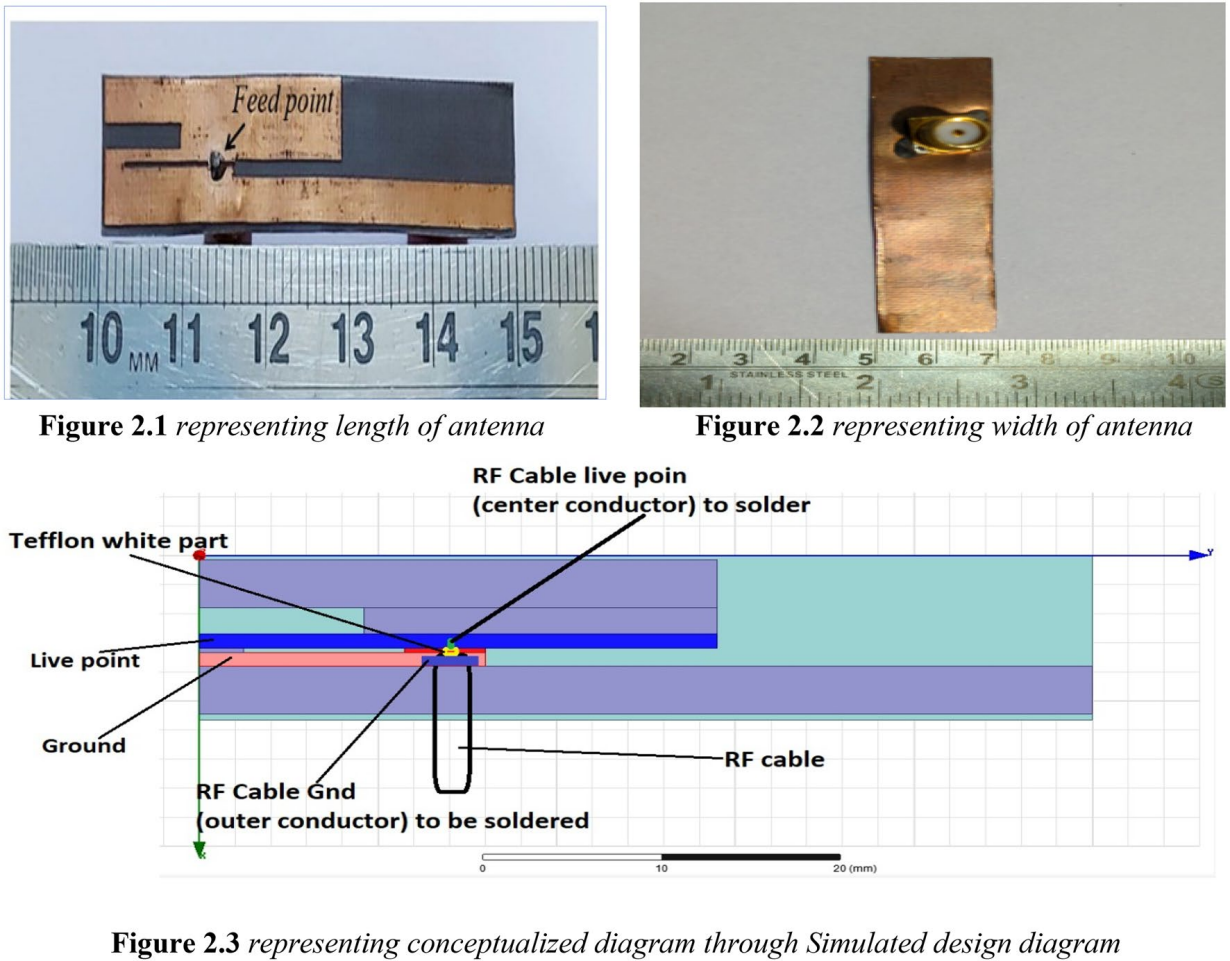
Fabricated antenna design with the SMA connector soldered and Simulated figure.

The proposed antenna presented in our research work acts as a dipole antenna with two elements, both acting as a ground for each other. The antenna produces an omnidirectional radiation pattern and is discussed in detail in Section. The fabricated antenna design is shown in Fig. 2. The antenna was fabricated using the popular photolithography process on one side of the RT-5880 substrate. The measured and simulated results are discussed in section “Simulated and measured results”.

## Simulated and measured results

The Proposed antenna was simulated using CST Microwave Studio as per the designing mentioned and discussed in above figures. Different parameters of simulated design and actual fabricated antenna has been compared and they found to be in good agreement.

**Figure 3:**
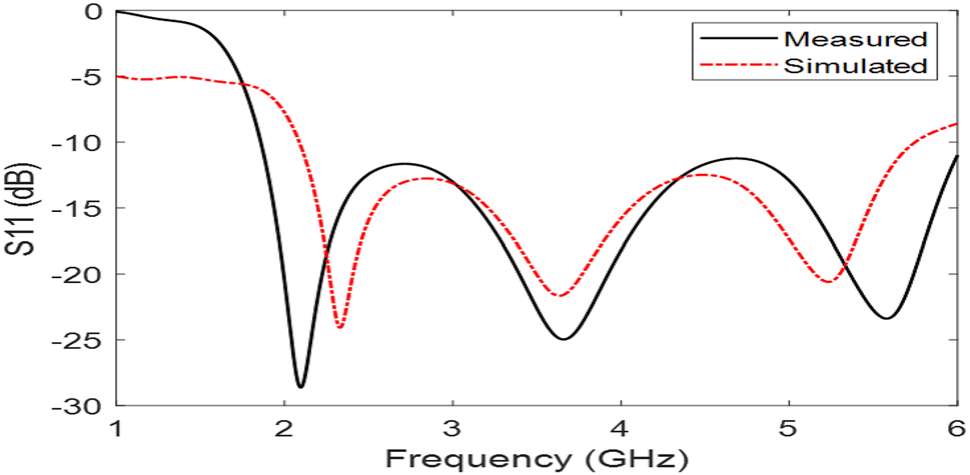
Measured and simulated S11 for the proposed antenna design.

**Figure 4:**
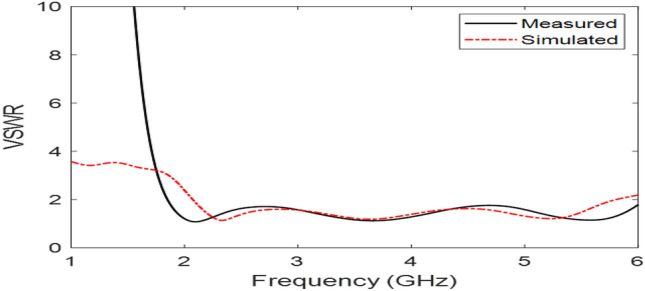
Measured and simulated voltage standing wave ratio (VSWR).

**Figure 5:**
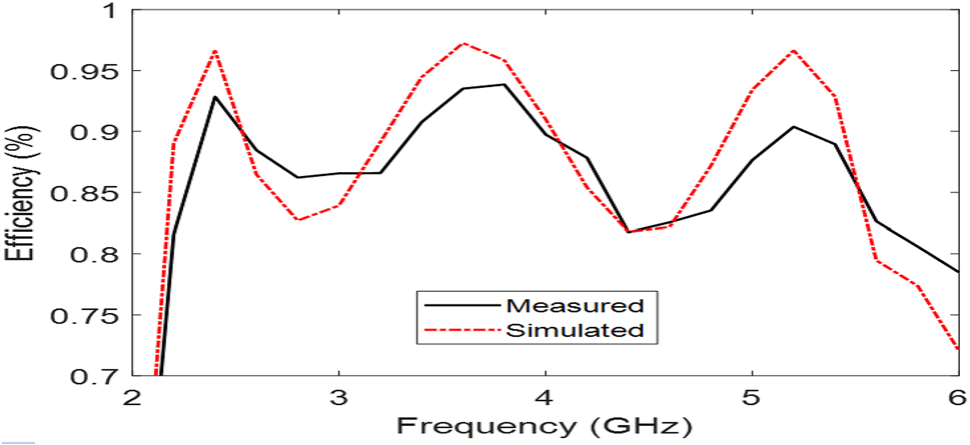
Measured and simulated total efficiency of the antenna.

**Figure 6:**
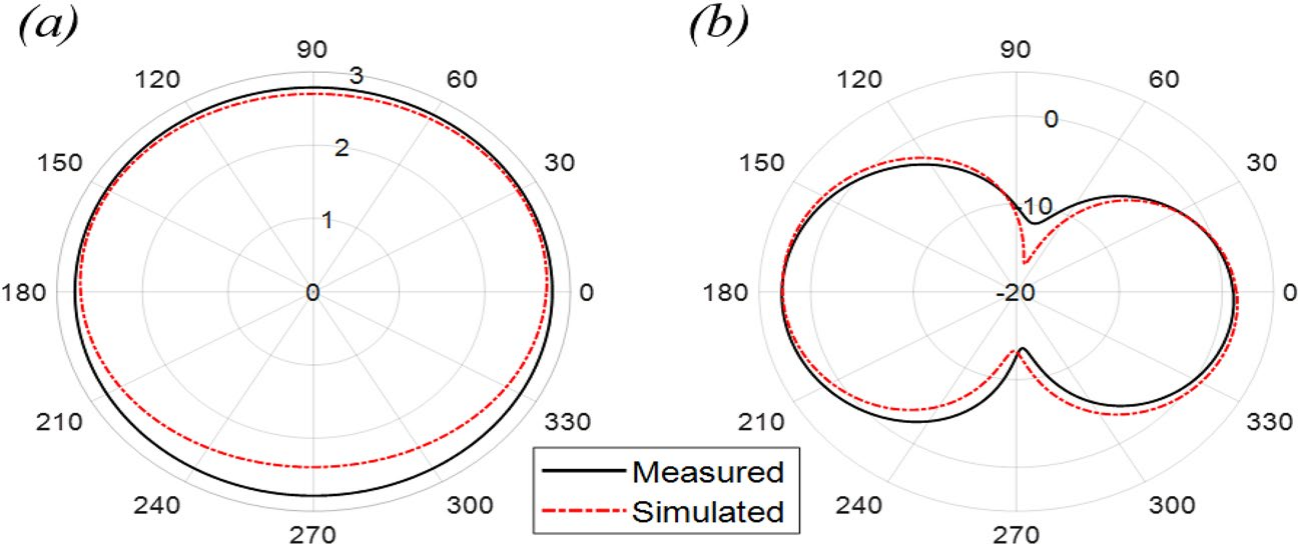
Measured and simulated radiation pattern at 2.4 GHz in the (**a**) azimuth and (**b**) elevation plane.

**Figure 7:**
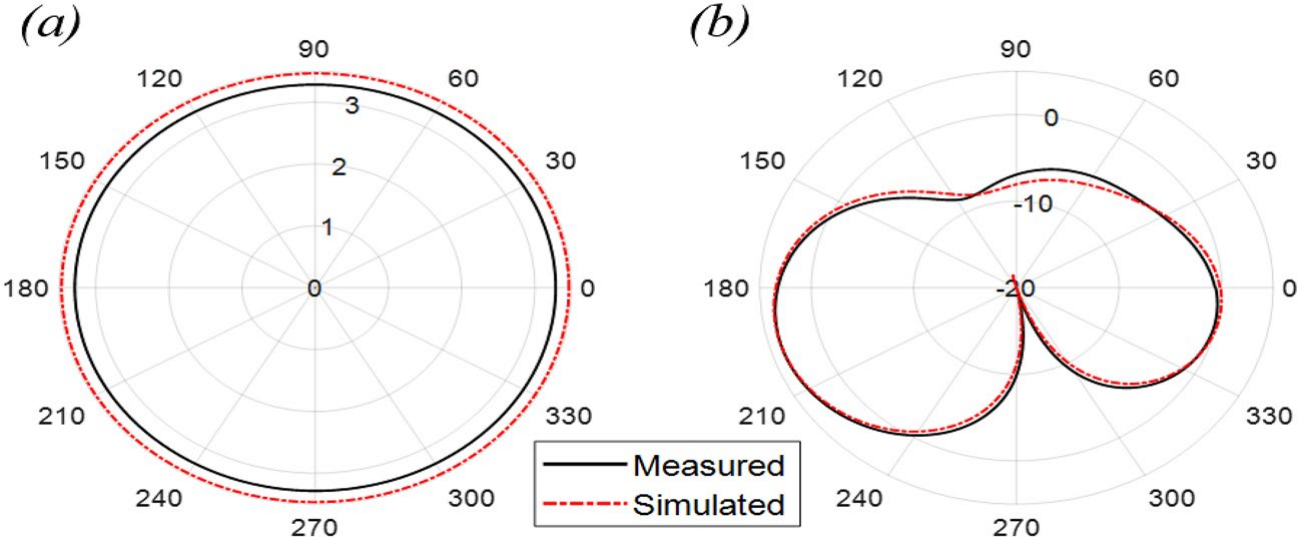
Measured and simulated radiation pattern at 3.5 GHz in the (**a**) azimuth and (**b**) elevation plane.

**Figure 8:**
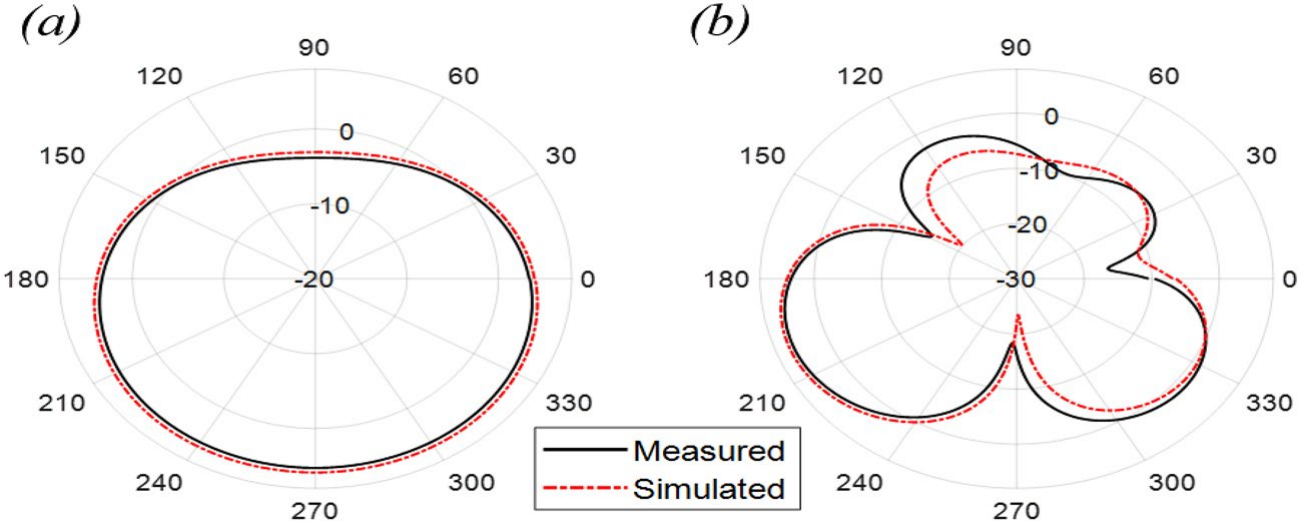
Measured and simulated radiation pattern at 5.5 GHz in the (**a**) azimuth and (**b**) elevation plane.

1. *Impedance bandwidth (S*_*11*_*)* The measured and simulated S-Parameter is shown in Fig. 3. The antenna has a wideband of 1.8 to 6 GHz, showing an approximately 108% bandwidth. This 108% bandwidth can be seen mathematically as – $Impedance BW=\frac{6-1.8}{3.9}\times 100$ = 108%, where fh = 6 GHz, fl = 1.8 GHz and fc = 3.9 GHz. By observing the curve presented over here we can see that the antenna has 3 different notches. The antenna is shown to have three bands, focusing at 2.4 GHz, 3.6 GHz, and 5.5 GHz which serves all major applications such as GPS, LTE, Wi-Fi, 5G, Wi-Max, etc. The measured results have a good agreement with the simulated results.
2. *VSWR* The Voltage standing wave ratio can be seen with the measured VSWR as shown in Fig. 4 here we can notice that the range of frequencies over which our antenna is giving less than -10 dB return loss the VSWR value is lying down between 1 to 2 which is desirable as well.
3. *Efficiency* The antenna demonstrates a strong measured efficiency of greater than 80%, with more than 90% efficiency for the three frequency bands. The measured and simulated efficiency is compared in Fig. 5.
4. *Radiation pattern* The antenna demonstrates an omnidirectional nature, agreeing to a general monopole antenna design. The measured radiation pattern at 2.4 GHz, 3.5 GHz, and 5.5 GHz are shown in Figs. 6, 7 and 8, respectively.

The antenna offers a positive gain throughout the required frequency band which increases as we go up higher in the frequency band. The half power beamwidth (HPBW) of the antenna is 125° in the elevation plane and a full 360° in the azimuth plane at 2.4 GHz; 84° in the elevation plane and 360° in the azimuth plane at 3.5 GHz; and 75° in the elevation plane and 270° in the azimuth plane at 5.5 GHz. The co-polarized results were found below -30 dB, and hence are not included here.

**Figure 9:**
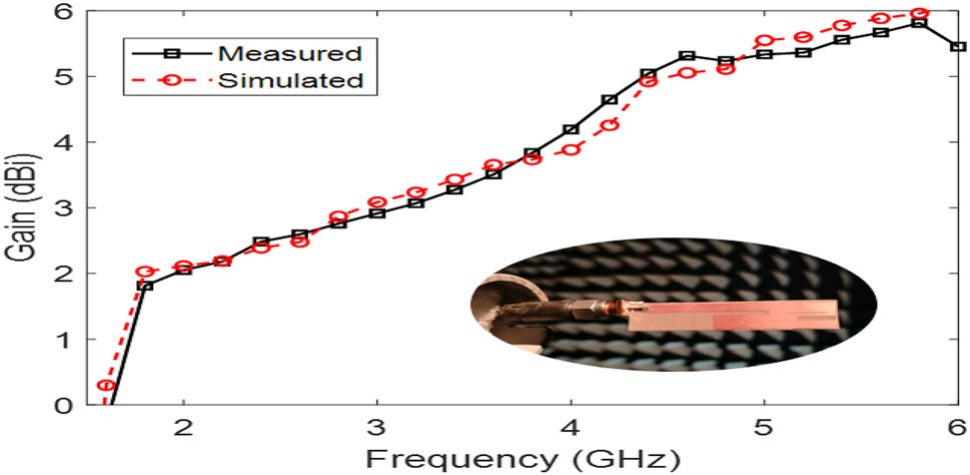
Measured and simulated gain of the proposed antenna (Inset: fabricated antenna inside the anechoic chamber).

**Figure 10:**
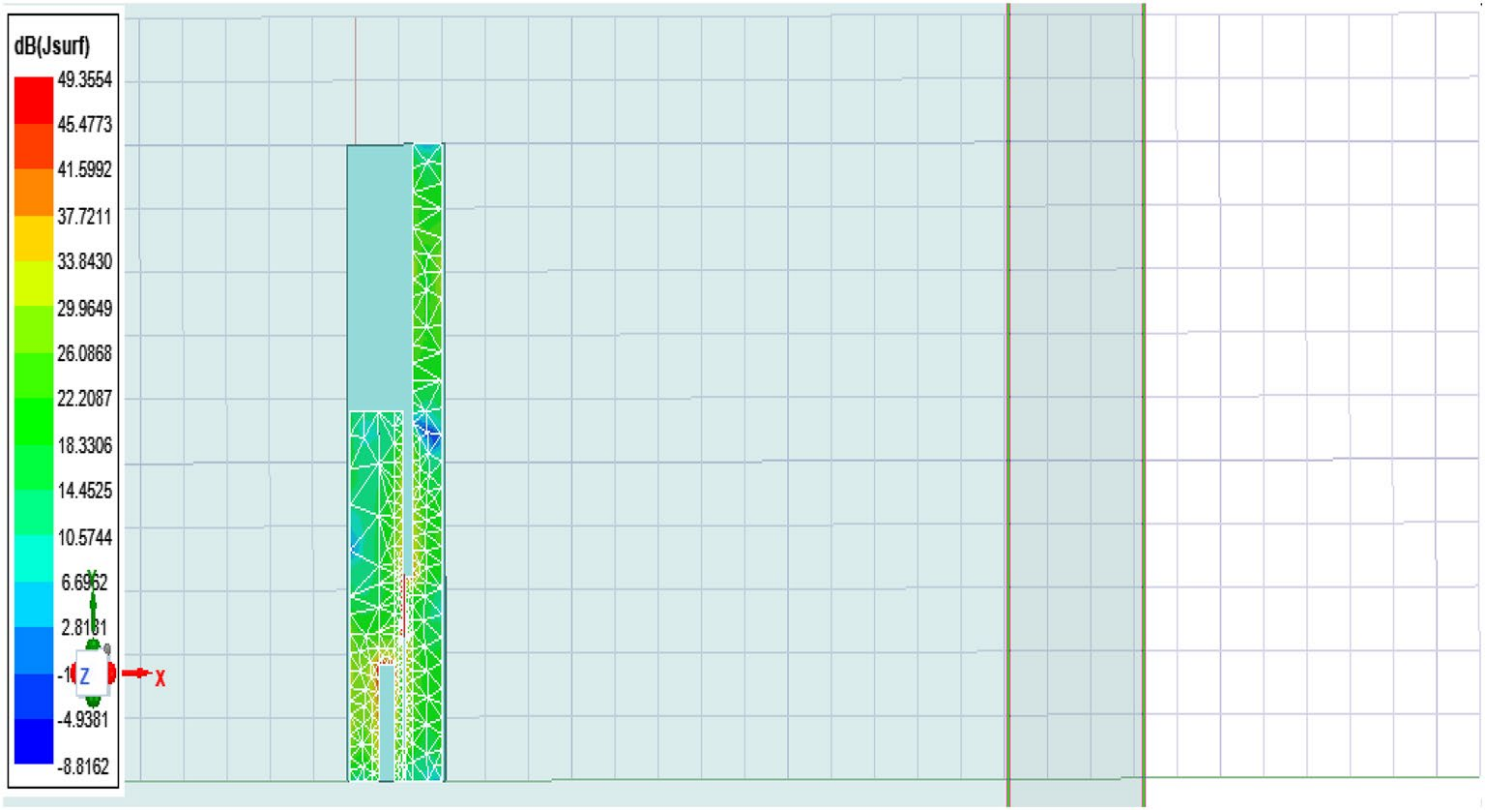
Current distribution at 2.4 GHz.

**Figure 11:**
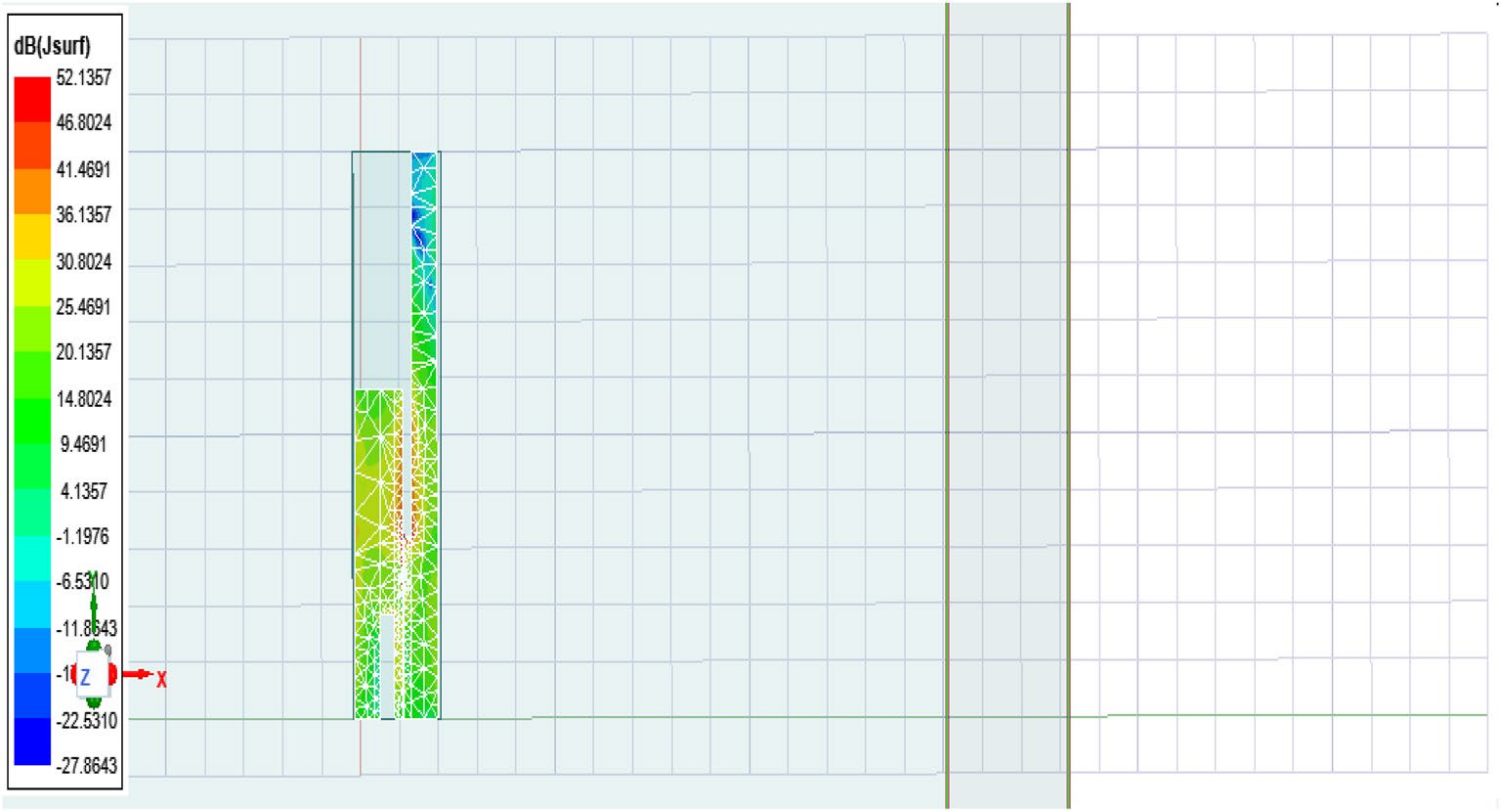
Current distribution at 3.5 GHz.

**Figure 12:**
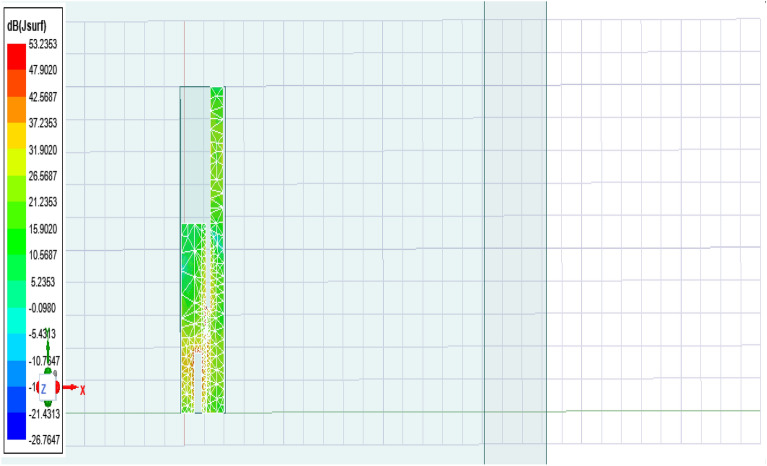
Current distribution at 5.5 GHz.

(5) *Gain* The measured and simulated gain for the antenna is shown in Fig. 9. The desirable gain value for antenna is positive value and should be in synchronization with other parameters. Here we can see the gain value for the accepted range is from 0.8 to 6 dB which is very good seeing the other parameters value also.
(6) *Current distribution *The current distribution on various resonant frequencies has been observed through Figs. 10, 11 and 12. We observed that this designed antenna will leads to better antenna efficiency as current distribution shows less losses through surface current and spurious radiation attached to it.

This presented folded dipole antenna achieves high radiated gains in its operational band through effective impedance matching, broadband performance, constructive interference of currents, and overall design efficiency. The combination of these elements results in an antenna that not only has a radiating pattern that covers all directions but also achieves greater amplification within its designated frequency range.

## Conclusion

This paper presented a miniaturized microstrip antenna design operating at a wideband of 1.8 to 6 GHz frequency band. The antenna presents a strong gain at the three frequency bands at 2.4, 3.5 and 5.5 GHz frequency bands. The antenna operates as a dipole antenna and offers an omnidirectional radiation pattern. The antenna is fed using an SMA connector and has a 50 Ω impedance match. The antenna has a measured gain of 0.8 to 6 dBi throughout the frequency band. The measured results have a very good agreement with the simulated results. Based on the observed results, and the literature survey presented in Section I, the proposed antenna is believed to be suitable for several fifth-generation mobile communication and IoT based applications the main focus of designing this presented design is to make an antenna which could utilized the sub 6 GHz frequency band which is the prominent part of high-speed communication 5G services.

## Data Availability

The datasets used during the current study are available from the corresponding author on reasonable request.
